# Hypoxia–Immune-Related Gene SLC19A1 Serves as a Potential Biomarker for Prognosis in Multiple Myeloma

**DOI:** 10.3389/fimmu.2022.843369

**Published:** 2022-07-25

**Authors:** Wenjin Li, Peng Yuan, Weiqin Liu, Lichan Xiao, Chun Xu, Qiuyu Mo, Shujuan Xu, Yuchan He, Duanfeng Jiang, Xiaotao Wang

**Affiliations:** ^1^ Department of Hematology, Pingxiang People’s Hospital, Pingxiang, China; ^2^ Department of Hematology, Affiliated Hospital of Guilin Medical University, Guilin, China; ^3^ Department of Hematology, Second Affiliated Hospital of Hainan Medical College, Haikou, China; ^4^ Department of Hematology, The Second Affiliated Hospital of Guilin Medical University, Guilin, China; ^5^ Department of Hematology, The Third Xiangya Hospital, Central South University, Changsha, China

**Keywords:** multiple myeloma, hypoxia, immune, SLC19A1, prognosis

## Abstract

**Background:**

Multiple myeloma (MM) remains an incurable malignant tumor of plasma cells. Increasing evidence has reported that hypoxia and immune status contribute to the progression of MM. In this research, the prognostic value of the hypoxia–immune-related gene SLC19A1 in MM was evaluated by bioinformatics analysis.

**Method:**

RNA-sequencing (RNA-seq) data along with clinical information on MM were downloaded from the Gene Expression Omnibus (GEO) database. Consistent clustering analysis and ESTIMATE algorithms were performed to establish the MM sample subgroups related to hypoxia and immune status, respectively, based on the GSE24080 dataset. The differentially expressed analysis was performed to identify the hypoxia–immune-related genes. Subsequently, a hypoxia–immune-gene risk signature for MM patients was constructed by univariate and multivariate Cox regression analyses, which was also verified in the GSE4581 dataset. Furthermore, the mRNA expression of SLC19A1 was determined using qRT-PCR in 19 MM patients, and the correlations between the genetic expression of SLC19A1 and clinical features were further analyzed.

**Result:**

A total of 47 genes were identified as hypoxia–immune-related genes for MM. Among these genes, SLC19A1 was screened to construct a risk score model that had better predictive power for MM. The constructed prognostic signature based on SLC19A1 was verified in the GSE4581 dataset. All independent prognostic factors (age, β_2_-microglobulin, LDH, albumin, MRI, and gene risk score) were used to develop a nomogram that showed a better performance for predicting the survival probability of MM patients for 1–5 years. Furthermore, SLC19A1 was highly expressed in newly diagnosed and relapsed MM patients, and high expression of SLC19A1 is correlated with higher bone marrow aspiration plasma cells and β_2_-microglobulin levels in MM patients.

**Conclusion:**

In conclusion, our results suggest that SLC19A1 is aberrantly expressed in MM and highly expressed SLC19A1 might be a biomarker correlated with inferior prognosis. More importantly, we identified SLC19A1 as a hypoxia–immune-related gene in MM. Future functional and mechanistic studies will further clarify the roles of SLC19A1 in MM.

## Introduction

Multiple myeloma (MM) is a plasma cell malignancy and remains incurable (1, 2). The main clinical manifestations of MM are renal insufficiency, hypercalcemia, bone marrow hematopoietic suppression, osteolytic bone lesions, and bone pain (3, 4). Diagnostic and prognostic biomarkers are used for predicting clinical outcomes and helping therapy selection and optimization so that MM patients can benefit from specific therapies (5, 6). Precise medical treatment of MM will require appropriate supporting diagnostic tests to detect the targetable lesions (5, 7); however, assay cost and availability remain major obstacles. This emphasizes the need to identify new molecules to provide prognostic biomarkers and/or therapeutic targets.

Hypoxia is one of the hallmarks of cancer, which is significantly associated with the occurrence and development of cancer (8). Hypoxia/oxygen sensing signal plays a vital role in the regulation of tumor progression (9–11). Previous studies have proven that the demethylases, KDM6A, JMJD3, JMJD6, and LSD1, have an affinity for oxygen, indicating their important significance in oxygen sensing and hypoxic reprogramming (12–14). In a variety of cancer models, the adaptation of tumor cells to the imbalance of oxygen demand and supply is related to inferior clinical outcomes (9, 15, 16). Through regulating gene and protein expression, hypoxia mediates genetic instability, malignant progression, and resistance to standard therapies (17).

In physiological immunological niches, such as the bone marrow, physiological hypoxia controls adaptive and innate immunity through transcriptional changes driven by the hypoxia-inducible factor (HIF) (18–20). On the contrary, in pathological immunological niches, such as tumors, pathological hypoxia can drive disease development and tissue dysfunction, *via* leading to immune cell imbalance (18). Through regulating the immunosuppressive effect, hypoxia also plays a vital role in anticancer immune responses (17). HIF signaling in liver cancer cells and innate immune cells together is conducive to the recruitment and maintenance of protumorigenic immune cells, inhibiting antitumorigenic immune cells and promoting immune evasion (21).

**Figure 1:**
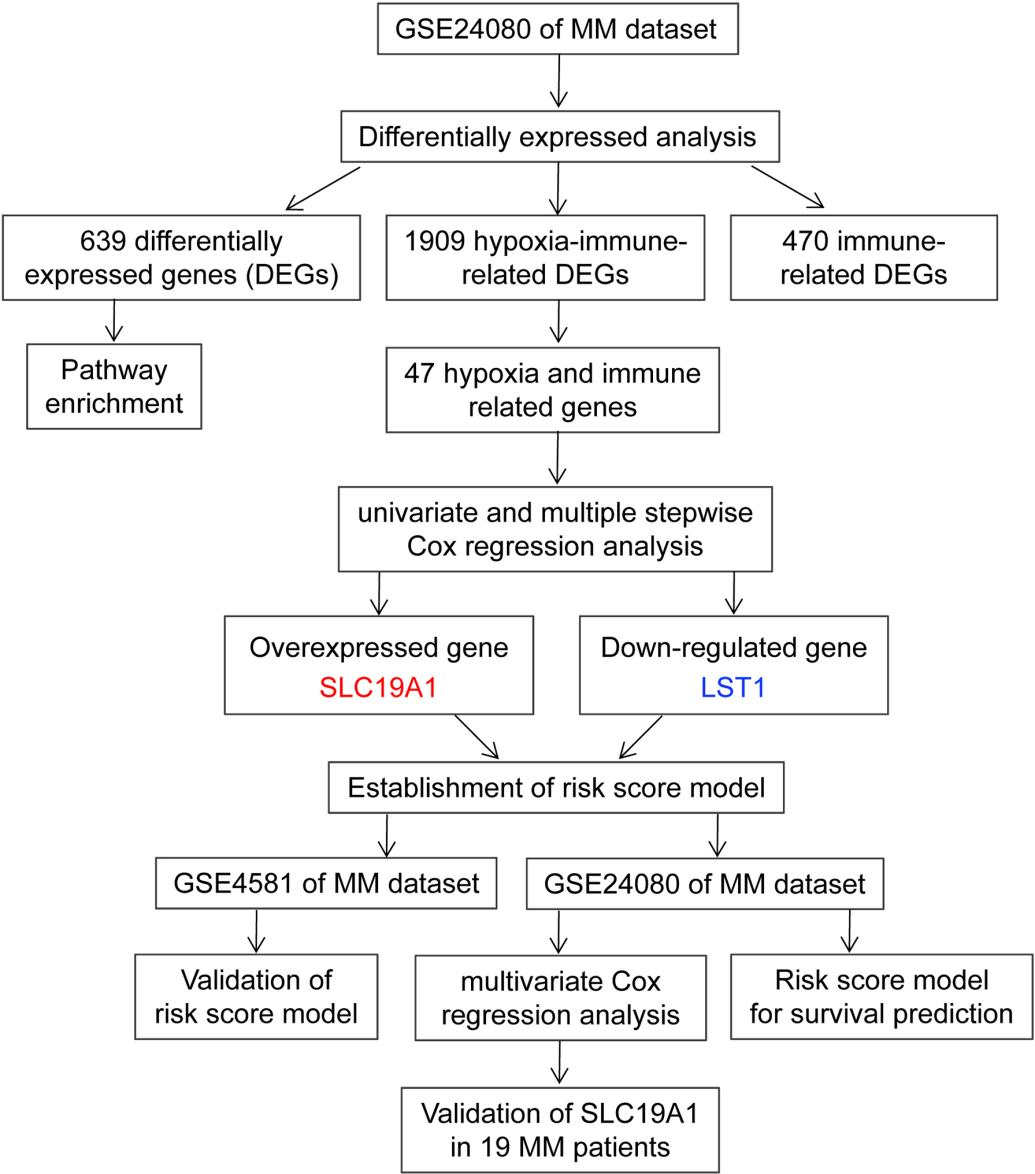
Overall design to screen and validate the hypoxia–immune-related gene SLC19A1 for multiple myeloma.

The reduced folate carrier (RFC, SLC19A1), thiamine transporter-1 (THTR1, SLC19A2), and thiamine transporter-2 (THTR2, SLC19A3) evolved from the same solute carrier family. SLC19A1 transports folates and SLC19A3 mediates intestinal thiamine absorption. SLC19A1 and SLC19A2 deliver their substrates to systemic tissues. SLC19A2 and SLC19A3 transport thiamine (22). THTR2 (SLC19A3) mRNA levels are downregulated in breast cancer, and the sensitivity to adriamycin was increased in the breast cancer cell line when transfected with SLC19A3 (23, 24). The SLC19A3-transfected cells were more sensitive to doxorubicin but not to paclitaxel or methotrexate (23). SLC19A1 is the primary means of cellular uptake for antifolate chemotherapeutic drugs;. therefore, the antifolate membrane transport of SLC19A1 is considered to be an important factor affecting antitumor activity (25). The reduced SLC19A1 plays a vital role in the transport of 5-methyltetrahydrofolate into the cells, and its lower expression has been related to the drug resistance of folate antagonists (26). In addition, SLC19A1 is the major transporter of cyclic dinucleotides (CDNs) into cells and has implications for the immunotherapeutic treatment of cancer (27–29). However, SLC19A1 has been identified but not yet studied for its impact on prognostic prediction or function in MM. In the present research, we tried to identify a group of prognostic genes related to hypoxia and immunity and construct a risk score model. Furthermore, the hypoxia–immune-related risk groups and several vital clinical parameters were included in a nomogram, which contributes to risk stratification and prognosis. The overall design of this research is shown in **Figure 1**.

## Methods and Materials

### Data Collection

In our study, the gene expression profiles and corresponding clinical information of MM were retrieved from the Gene Expression Omnibus (GEO) (https://www.ncbi.nlm.nih.gov/geo/) database (GSE24080 and GSE4581) (30–33). The GSE24080 dataset was composed of 554 MM patients. The GSE4581 dataset was utilized as a validation set that included 414 MM samples to verify the prognostic risk signature.

### Acquisition of Hypoxia-Related Differentially Expressed Genes

The 198 hypoxia-related genes were downloaded from the Molecular Signatures Database (MSigDB, https://www.gsea-msigdb.org/gsea/msigdb/) database. To determine the hypoxia status (hypoxia^high^ and hypoxia^low^) of MM samples from the GSE24080 dataset, a consistent clustering analysis was conducted based on the 198 hypoxia-related genes using the R package ConsensusClusterPlus. The similarity distance between MM samples was computed by the Euclidean distance, and the optimal number of the clusters was determined by the cumulative distribution function (CDF). The different overall survival (OS) among the identified different clusters was detected by the K-M curve analysis using the survival package in R.

### Differentially Expressed Analysis

*p* < 0.05 and |log_2_ Fold Change| ≥0.5 were set as the cutoffs to identify hypoxia-related differentially expressed genes (DEGs) between the hypoxia^high^ and hypoxia^low^ group using the limma package in R. The same selection standard was utilized to identify the immune-related DEGs and hypoxia–immune-related DEGs.

### Functional Enrichment Analysis of Hypoxia-Related DEGs

For the hypoxia-associated DEGs, the functions and pathway enrichment were investigated by the gene ontology (GO) annotation and Kyoto Encyclopedia of Genes and Genomes (KEGG) pathway analysis utilizing the R package clusterProfiler. The cutoff criterion was the false-discovery rate (FDR) of <0.05.

### Selection of Immune-Related DEGs

The immune scores of MM samples were assessed by the ESTIMATE algorithm using the R package estimate. The optimal cutoff of the immune score calculated by the surv cutpoint algorithm of the R package survival was used to classify MM samples into two groups: immune^high^ group and immnue^low^ group.

### Identification of Hypoxia–Immune-Related Genes

To explore whether there is an internal relationship between immunity and hypoxia in the pathogenesis and development of MM disease, hypoxia and immune statuses were further combined into a two-dimension indicator that separated MM samples into low hypoxia and high immune groups (hypoxia^low^/immune^high^), high hypoxia and low immune groups (hypoxia^high^/immune^low^), and a mixed group including high hypoxia and the high immune group as well as low hypoxia and low immune group (hypoxia^high^/immune^high^ + hypoxia^low^/immune^low^). Survival analysis of these three groups was executed by K-M plots, and the *p* < 0.05 was considered statistically significant.

Moreover, the DEGs between hypoxia^low^/immune^high^ and hypoxia^high^/immune^low^ groups were screened using the “limma” R package, with *p*-value <0.05 and |log2FC|≥0.5. Also, the volcano plot and heatmap were applied to the visualization of DEGs’ expression. The key hypoxia–immune-related genes were obtained by overlapping the DEGs (hypoxia^low^/immune^high^ VS hypoxia^high^/immune^low^) and hypoxia/immune-related DEGs.

### Constructed Prognostic Risk Signature Associated With Hypoxia and Immune Statuses

Firstly, the MM samples in the GSE24080 dataset were randomly stratified into an internal training set (*n* = 388) and an internal testing set (*n* = 166) at a ratio of 7:3. In the training set, K-M curve and univariate Cox regression analyses were operated to screen genes related to the OS of MM (*p* < 0.05), which were regarded as prognosis-based genes. Multivariate COX regression analysis was then used to identify the most predictive prognostic genes. The risk score was calculated according to the gene expression and coefficient value of samples. The formula was as follows:


$$\text{Risk score}\;=\;e^{\text{sum }\left(\text{each gene's expression levels}\;\times \;\text{corresponding coefficient}\right)}\;/\;e^{\text{sum }\left(\text{each gene's mean expression levels }\times \text{ corresponding coefficient}\right)}$$

Furthermore, the risk score was determined by the predicted Cox ph algorithm of the R survival package, and the patients were classified into a high- and low-risk group based on the median risk score. A comparison of the survival between the two risk groups was tested by the K-M curve analysis. The receiver operating characteristic (ROC) analysis was employed to measure the predictive power of the risk model using the survival-ROC package in R. In addition, the areas under the curve (AUC) of each of the resulting ROC curves were used to assess the model’s discrimination.

### Independent Prognostic Analysis and Construction of a Nomogram

Stepwise, univariate and multivariate COX regression analyses were performed to assess whether clinicopathological features (PROT, AGE, SEX, PACE, B2M, CPR, CREAT, LDH, ALB, HGB, ASPC, BMPC, MRI, CPS1, CPR1, FLC, lgA, lgD, lgG, nonsecretory, NSE) and gene risk score were independent prognostic factors for MM patients (*p* < 0.05). Hazard ratios (HRs) estimated from Cox models were reported as relative risks with corresponding 95% confidence intervals (CIs). HRs >1 were considered a risk factor, and HRs <1 were considered a protective factor. All identified independent prognostic factors were then included to construct a nomogram using the R package rms to predict the survival probability. Concordance indexes (C-index) and calibration curves were used to assess the discrimination and calibration of the nomogram.

### Real-Time Quantitative Polymerase Chain Reaction

Bone marrow (BM) samples derived from healthy candidate donors (*n* = 5), newly diagnosed MM patients (*n* = 13), and relapsed MM patients (*n* = 6) were obtained with consent at Pingxiang People’s Hospital and the Third Xiangya Hospital of Central South University (CSU) during the period 2018–2020. The experimental protocols were approved by the ethical committee of CSU. BM mononuclear cells were isolated with density gradient separation using Ficoll-Hypaque (Sigma-Aldrich, St Louis, MO). CD138^+^ cells were enriched using the Miltenyi microbead separation system (Miltenyi BioTech, Auburn, CA). Bone marrow processing was done within 2 h after collection. The clinical information of the specimens is listed in **Supplementary Table S1**. RNA was isolated from 1 × 10^6^ cells using TRIzol reagent (Vazyme, Nanjing, China). Then, 2 μg total RNA was reverse transcribed into cDNA with the HiScript III RT SuperMix (Vazyme). cDNA was amplified in a total volume of 20 μl using the SYBR qPCR Master Mix (Vazyme) and the amplification was performed by LightCycler 480 (Roche, Burgess Hill, UK) with specific primers (**Table 1**). The Gene expression levels were calculated using the 2^−ΔΔCt^ method (35).

**Table 1:** Characteristics of gene-specific real-time PCR assays.

Gene/Accession No.	Mer	Amplicon (bp)	Conc. (nM)	Resources
**SLC19A1 [GenBank:NM_001352512.2]**				Primer Express^®^
Forward 5′-CTTTGCCACCATCGTCAAGACC-3′	22	115	250	
Reverse 5′-GGACAGGATCAGGAAGTACAC -3′	22		250	
**β-Actin [GenBank: NM_001101.5]**				Jiang et al. (34)
Forward 5′-GGACTTCGAGCAAGAGATGG-3′	20	234	250	
Reverse 5′-AGCACTGTGTTGGCGTACAG-3′	20		250	

Source: J Transl Med. May 17 2021; 19(1):211.

### Statistical Analysis

Statistical analysis was conducted using the R software. Comparisons in proportions of variables between two groups were analyzed using the *χ* ^2^ test. An unpaired *t*-test or the Mann–Whitney *U* test was performed for the differences between continuous variables. Unless otherwise stipulated, the *p* < 0.05 was considered statistical significance.

## Results

### Identification of Hypoxia-Related Genes for MM

**Figure 2:**
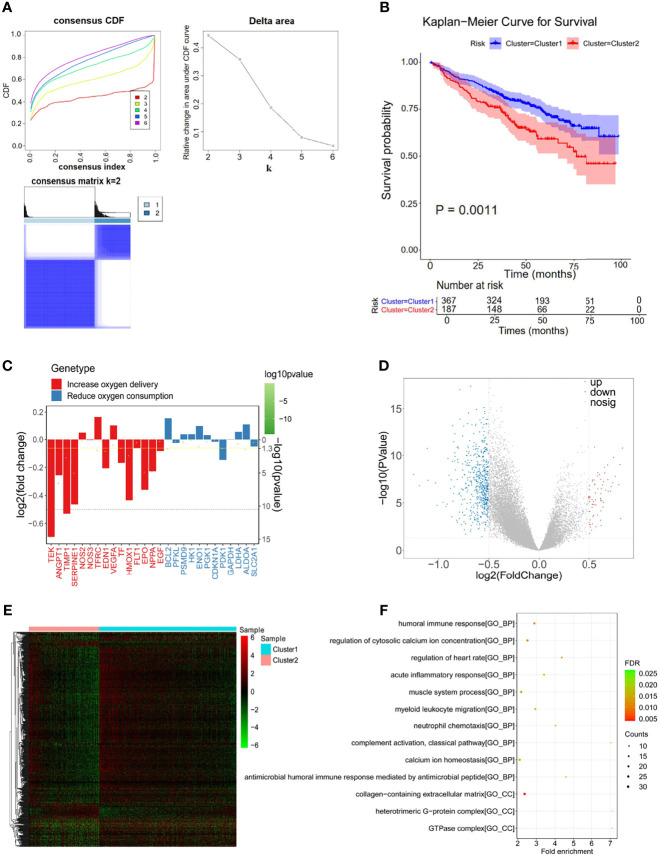
Differentially expressed analysis of hypoxia-associated 639 genes for multiple myeloma (MM). **(A)** The Euclidean distance was used to calculate the similarity distance between MM samples, and the optimal number of the clusters was determined by the cumulative distribution function (CDF). **(B)** Kaplan–Meier analysis of the different clusters to predict the survival of the patient. Upper: Kaplan–Meier curve of the overall survival between clusters 1 and 2 groups. Lower: the number of patients at risk in clusters 1 and 2 groups at different time points. **(C)** Identified the hypoxia status between the two clusters by analyzing the target gene expression changes of the HIF-1 signaling pathway. **(D**, **E)** In total, 639 hypoxia-related differentially expressed genes (DEGs) were identified with hierarchical cluster analysis for MM. **(F)** Functional enrichment analysis of the 639 DEGs.

Hypoxia is well known as a critical hallmark of the tumor microenvironment (36). With the expression matrix of 198 hypoxia-related genes from the MSigDB database, the Euclidean distance was calculated between two cases in the GSE24080 dataset to identify the hypoxia status in the tumor environment of MM patients. As shown in **Figure 2A**, *k* = 2 was found to be more optimal and classified MM samples into two groups with less correlation: clusters 1 and 2. The result of the survival comparison analysis suggested that cluster 2 patients presented worse OS than cluster 1 patients (*p* = 0.0011, **Figure 2B**). The target gene expression changes of the HIF-1 signaling pathway were analyzed to identify the hypoxia status between the two clusters. Nine of 15 genes (60%) associated with the process of increasing oxygen delivery were overexpressed in cluster 1, while 2 of 12 genes (16.7%) associated with the process of reducing oxygen consumption were increased in cluster 2 (**Figure 2C**). Taken together, we uncovered that patients in clusters 1 and 2 were classified as hypoxia^low^ and a hypoxia^high^, respectively. Moreover, we identified 639 DEGs between clusters 2 and 1 that were considered hypoxia-related DEGs for MM, containing 71 upregulated and 568 downregulated genes (**Figures 2D, E**; **Supplementary Table S2**). The results of functional enrichment analysis demonstrated that those DEGs were significantly enriched in the immune-related terms and pathways, including humoral immune response, acute inflammatory response, and myeloid leukocyte migration (**Figure 2F**; **Supplementary Figure S1**).

### Identification of Immune-Related Genes for MM

**Figure 3:**
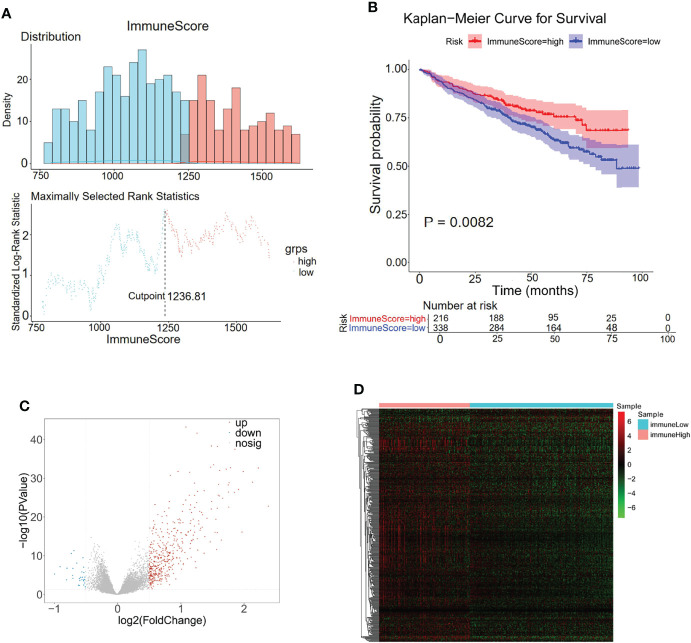
Differentially expressed analysis of immune-related 470 genes for MM. **(A)** The distributions of immune scores across multiple myeloma samples in the GSE24080 datasets. **(B)** Kaplan–Meier analysis of the different immune scores to predict patient survival. **(C**, **D)** In total, 470 immune-related differentially expressed genes were identified with hierarchical cluster analysis for MM.

Considering the importance of the immune response in the MM tumor environment, the immune status was identified by ESTIMATE. The MM samples were clustered into high and low immunity groups based on the calculated median immune score (**Figure 3A**), which showed a significant difference in survival between the two groups (**Figure 3B**). Patients with high immune scores represented a higher survival rate than those with low immune scores (*p* = 0.0082, **Figure 3B**). Moreover, 443 genes were overexpressed in the immune^high^ group and 27 genes were overexpressed in the immune^low^ group, which were considered immune-related DEGs (**Figures 3C, D**; **Supplementary Table S3**).

### Identification of Hypoxia–Immune-Related Genes for MM

**Figure 4:**
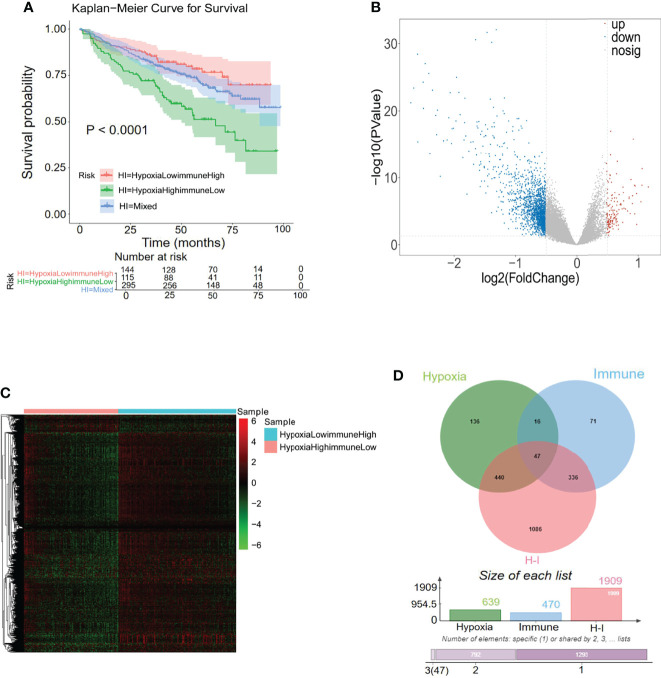
Differentially expressed analysis of hypoxia–immune-related 47 genes for MM. **(A)** Kaplan–Meier analysis of the different groups based on the hypoxia and immune status to predict patient survival. **(B, C)** In total, 1,909 hypoxia–immune-related differentially expressed genes (DEGs) were identified with hierarchical cluster analysis for MM. **(D)** Forty-seven hypoxia–immune-related genes were obtained from intersecting with 639 hypoxia-related DEGs, 470 immune-related DEGs, and 1,909 hypoxia–immune-related DEGs.

A previous study has reported the interaction between hypoxia and immune status in the MM tumor microenvironment (36). The above hypoxia and immune statuses were combined as a two-dimension indicator that split MM patients into three groups: hypoxia^high^/immune^low^ group, hypoxia^low^/immune^high^ group, and mixed group (hypoxia^high^/immune^high^ + hypoxia^low^/immune^low^). K-M curve analysis demonstrated a remarkably different survival rate among the three groups. As shown in **Figure 4A**, the patients in the hypoxia^low^/immune^high^ group exhibited the best survival rate, while patients in the hypoxia^high^/immune^low^ group exhibited the worst survival rate, manifesting the mutual effects of hypoxia and immune response on the development of MM (*p* < 0.0001). A total of 1,909 DEGs were selected between the hypoxia^high^/immune^low^ group and the hypoxia^low^/immune^high^ group, in which 177 genes were upregulated in the hypoxia^high^/immune^low^ group and 1,765 genes were upregulated in the hypoxia^low^/immune^high^ group (**Figures 4B, C** **)**. To obtain the hypoxia–immune-related genes, the above selected 1,909 hypoxia–immune-related DEGs, 639 hypoxia-related DEGs, and 470 immune-related DEGs were intersected to obtain 47 genes for subsequent study (**Figure 4D**; **Supplementary Table S4**).

### Establishment of Risk Signature Related to the Hypoxia and Immune Status for the Prognosis of MM

**Figure 5:**
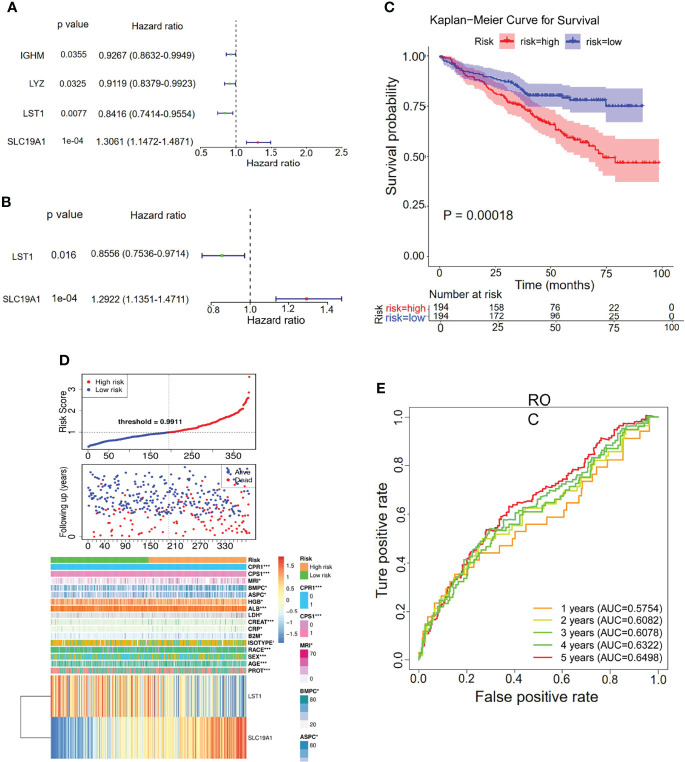
Univariate and multiple stepwise Cox regression analyses of the hypoxia–immune-related genes in MM. **(A**, **B)** Multivariate Cox regression analysis of the genes IGHM, LYZ, LST1, and SLC19A1. **(C)** Kaplan–Meier analysis of the risk score model based on SLC19A1 and LST1 to predict patient survival in the internal training set. Upper: Kaplan–Meier curve. Lower: the number of patients at different time points. **(D)** The risk score distribution and the survival status. According to the cutoff value, patients with multiple myeloma were divided into high- and low-risk groups. **(E)** Time-dependent ROC curves for the two-gene model to predict patient survival.

To explore the prognostic value of the hypoxia–immune-related genes in MM, we first performed the K-M curve and univariate Cox regression analyses in the internal training set. IGHM, LYZ, LST1, and SLC19A1 were identified as significantly associated with the overall survival of MM (**Figure 5A**; **Table 2**). Afterward, LST1 (*p* = 0.016, HR = 0.8556) and SLC19A1 (*p* = 1e−04, HR = 1.2922) were further identified by the multiple stepwise Cox regression analysis to generate a prognostic risk signature for MM (**Figure 5B**; **Table 3**). The individual-level gene risk score for a patient was calculated, and the cutoff risk score divided patients into a high- and low-risk group in the internal training set. Survival analysis indicated that patients in the high-risk group showed a poor prognosis compared to those in the low-risk group (**Figure 5C**). As shown in **Figure 5D**, the patients died with an increasing gene risk score. The AUC for survival rate was 0.5754 at 1 year, 0.6078 at 3 years, and 0.6498 at 5 years, implying a better prognostic ability for MM (**Figure 5E**). Moreover, SLC19A1 was overexpressed and LST1 was downregulated in the high-risk group (**Figure 5C**).

**Table 2:** Hypoxia-immune-related genes in K-M curve and univariate Cox regression analysis.

	Coefficient	HR (95% CI for HR)	Wald test	z	Cox *p*-value	KM *p*-value
IGHM	−0.0761	0.9267 (0.8632–0.9949)	4.42	−2.1025	0.0355	0.0449
LYZ	−0.0923	0.9119 (0.8379–0.9923)	4.57	−2.1386	0.0325	0.0433
LST1	−0.1725	0.8416 (0.7414–0.9554)	7.11	−2.666	0.0077	0.0062
SLC19A1	0.2671	1.3061 (1.1472–1.4871)	16.28	4.0347	1.00E-04	0.0031

**Table 3:** LST1 and SLC19A1 in the multiple stepwise Cox regression analysis.

	Coefficient	HR	*z*	*p*-value
LST1	−0.156	0.8556 (0.7536–0.9714)	−2.41	0.016
SLC19A1	0.2564	1.2922 (1.1351–1.4711)	3.88	0.0001

### Genetic Overexpression of SLC19A1 Is Associated With Poor Prognosis in MM

**Figure 6:**
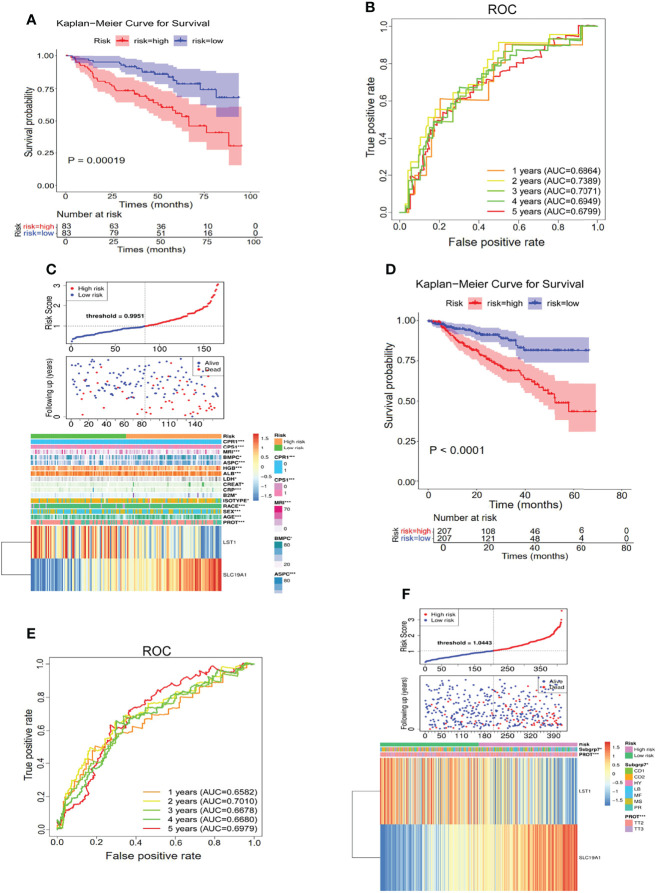
Genetic overexpression of SLC19A1 is correlated with poor prognosis in MM. **(A**, **D)** Kaplan–Meier analysis of the risk score model based on SLC19A1 and LST1 to predict patient survival in the internal testing set **(A)** and GSE4581 dataset **(D)**. **(B**, **E)** Time-dependent ROC curves for the two-gene model to predict patient survival in the internal testing set **(B)** and GSE4581 dataset **(E)**. **(C**, **F)** The risk score distribution and the survival status of the high- and low-risk groups in the internal testing set **(C)** and GSE4581 dataset **(F)**.

The constructed hypoxia–immune-gene prognosis signature based on SLC19A1 upregulation and LST1 downregulation was further validated in the internal testing set and GSE4581 dataset. Consistent with the findings in the internal training set, patients in the high-risk group showed shorter survival times than those in the low-risk group in both testing sets (**Figures 6A, D** **)**. Additionally, ROC analysis verified the results that the risk signature had good performance for prognosing MM patients (**Figures 6B, E** **)**. The detailed information on the distribution of risk score, survival status, and the two prognostic genes SLC19A1 and LST1 expression profiles in the two sets was displayed in **Figures 6C, F**. In conclusion, genetic overexpression of SLC19A1 is correlated with inferior prognosis in MM.

### Independent Prediction Capacity of the Risk Score Based on SLC19A1

**Figure 7:**
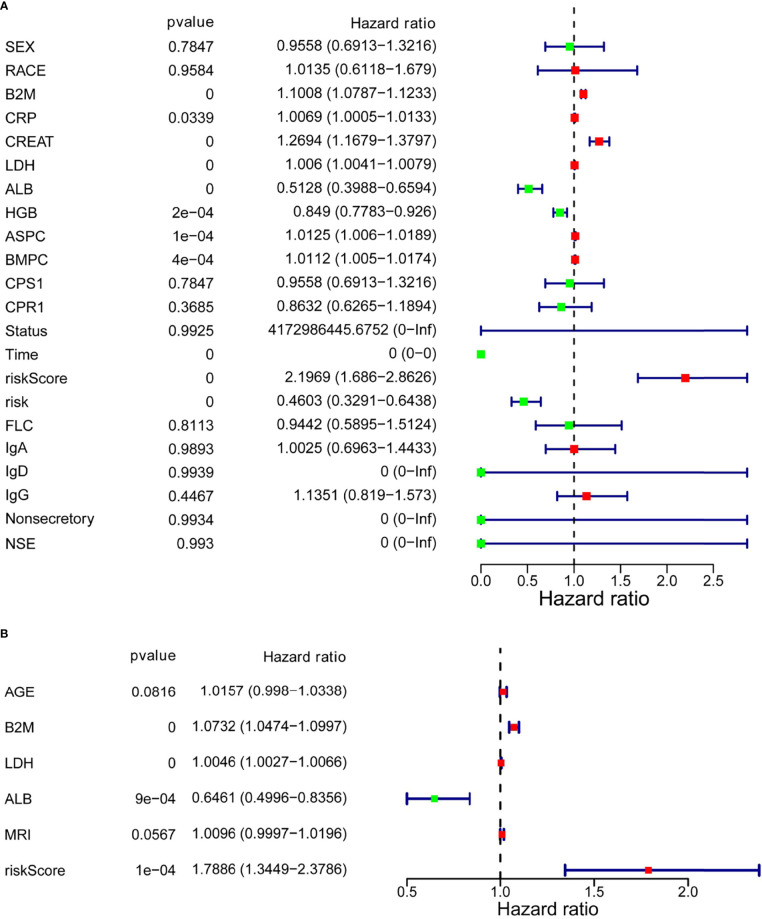
Multivariate Cox regression analysis of the SLC19A1 risk score and clinical risk parameters. **(A**, **B)** B2M, LDH, ALB, and SLC19A1 risk scores were markedly correlated with the overall survival of patients with multiple myeloma.

**Figure 8:**
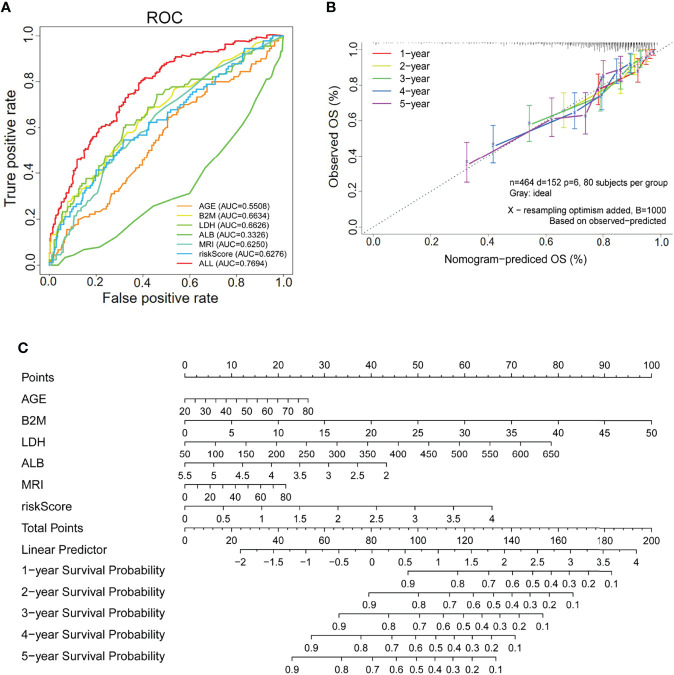
The SLC19A1 risk score and clinical risk parameters with multivariate Cox regression analysis. **(A)** ROC curves are utilized to compare the predictive performance for AGE, B2M, LDH, ALB, MRI, riskScore, and ALL to predict patient survival. **(B)** The calibration curves for predicting 1-, 2-, 3-, 4-, and 5-year overall survival. **(C)** Establishment of a nomogram for prediction of 1-, 2-, 3-, 4-, and 5-year survival of MM patients.

Next, to determine whether the risk score based on SLC19A1 was an independent prognostic factor for MM patients, the univariate and multivariate Cox regression analyses were conducted in the GSE24080 and GSE4581 datasets. In the GSE24080 dataset, age-identified clinical prognostic factors (β_2_-microglobulin, C-reactive protein, creatinine, LDH, albumin, hemoglobin, ASPC, BMPC, and MRI) and gene risk score were obviously connected with OS by univariate Cox regression analysis (**Figure 7A**) (38). The result of multivariate Cox regression analysis suggested that β_2_-microglobulin, LDH, albumin, and gene risk score were independent prognostic factors for MM patients (**Figure 7B**). The prognostic signature contained all harvested independent factors that had better predictive power than alternative options (**Figure 8A**). In addition, age, β_2_-microglobulin, LDH, albumin, MRI, and gene risk score were used to construct a nomogram to predict the 1-, 2-, 3-, 4, and 5-year overall survival of MM patients. The calibration plot manifested that the nomogram showed a good agreement with the ideal model, suggesting the constructed nomogram had great performance (**Figures 8B, C** **)**.

### High SLC19A1 Expression Is Correlated With Risk Stratification, Bone Marrow Aspiration Plasma Cell Numbers, and β_2_-Microglobulin in MM Patients

**Figure 9:**
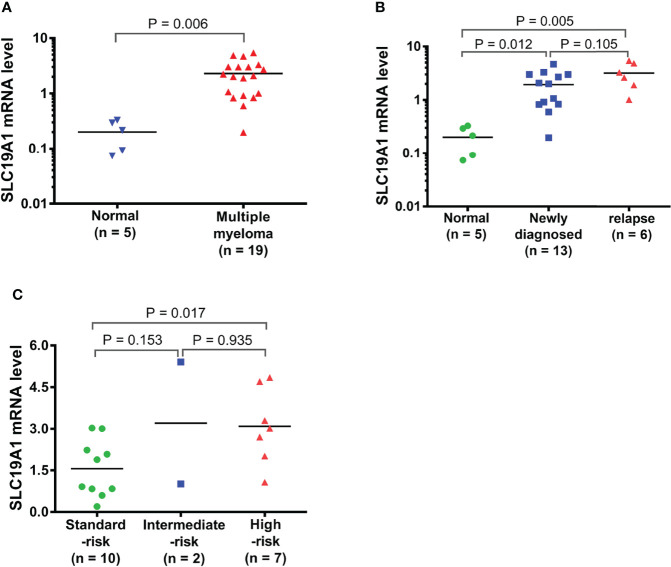
Expression of SLC19A1 in primary MM samples. **(A)** qRT-PCR analysis of SLC19A1 mRNA expression in MM patient samples (*n* = 19) and normal controls (*n* = 5). **(B)** SLC19A1 mRNA expression in newly diagnosed MM patients (*n* = 13), relapsed MM patients (*n* = 6), and normal controls (*n* = 5). **(C)** SLC19A1 mRNA expression in different risk stratifications. High risk defined as: del 17p, t(14;16), t(14;20), +1q, and del 1p. Intermediate risk defined as: t(4;14), del 13q, and hyperdiploidy. Standard-risk defined as: t(11;14), t(6;14), del 14q (IgH), and others.

The results indicated the expression levels of SLC19A1 in MM, including newly diagnosed and relapsed MM, were higher than those in normal control (**Figures 9A, B** **)**. Moreover, we found that patients with high-risk cytogenetics (39) had much higher SLC19A1 expression compared with standard-risk patients (*p* = 0.017, **Figure 9C**). To determine the correlation of SLC19A1 expression with clinical features in MM patients, we classified the patients into a low SLC19A1 expression group (SLC19A1^low^, *n* = 9) and a high SLC19A1 expression group (SLC19A1^high^, *n* = 10) based on the median SLC19A1 expression level. As shown in **Table 4**, the high expression of SLC19A1 was found to be correlated with a higher number of bone marrow aspirated plasma cells (*p* = 0.037). Moreover, marked differences were observed in the group of β_2_-microglobulin ≥5.5 mg/L (*p* = 0.029). However, no significant differences were observed in sex, age, albumin, C-reactive protein, LDH, creatinine, hemoglobin, cytogenetic abnormalities, and relapse MM patients between SLC19A1^high^ and SLC19A1^low^ patients (**Table 4**).

**Table 4:** Correlation of SLC19A1 expression with clinical and laboratorial parameters in MM patients.

Patient’s parameters	Total (median; range (*n* = 19))	SLC19A1 expression level		*p*-value
		High (% (*n* = 10))	Low (% (*n* = 9))	
Sex (male/female)	11/8	31.6	26.3	0.845
Age (≥65 years)	56 (43–76)	10.5	5.3	0.737
Albumin (<35 g/L)	32.7 (17.9–47.9)	42.1	26.3	0.515
β_2_-Microglobulin	4.0 (0.5–26.3)			
<3.5 mg/L		10.5	26.3	0.108
≥3.5 mg/L (<5.5 mg/L)		5.3	10.5	0.348
≥5.5 mg/L		36.8	10.5	0.037
C-reactive protein (≥8 mg/L)	6.9 (0.6–106.3)	31.6	10.5	0.096
LDH (≥230 IU/L)	218 (116–734)	36.8	15.8	0.110
Creatinine (≥176.8 µmol/L)	82.0 (5.6–488.0)	15.8	5.3	0.313
Hemoglobin (<100 g/L)	76 (42–147)	26.3	31.6	0.462
ASPC (≥30%)	45.2 (12.5–86.6)	42.1	15.8	0.029
Cytogenetic abnormalities		36.8	15.8	0.110
Relapse MM patients		21.1	10.5	0.405

MM, multiple myeloma; LDH, lactate dehydrogenase; ASPC, bone marrow aspiration plasma cells.

## Discussion

Hypoxia and HIFs are known to affect cancer cells by regulating the expression of many genes that control tumor cell metabolism, angiogenesis, tumor invasion, and metastasis (40, 41). Hypoxia in the tumor microenvironment regulates the function of immune cell effectors and plays a positive role in tumor development (18). Although there is a clear link between hypoxia and immunity (42, 43), fewer studies pay attention to the comprehensive molecular mechanism.

In the present study, we performed the differentially expressed analysis and Cox regression analysis to screen the potential hypoxia–immune-related genes and establish a gene-related risk score model to predict the overall survival (OS) of MM patients. Moreover, we included gene risk groups and clinical parameters in our nomogram to precisely predict patient survival. A total of 47 hypoxia–immune-related genes were analyzed by univariate and multiple stepwise Cox regression in our study, and the overexpressed gene SLC19A1 and the downregulated gene LST1 were identified as markedly correlated with the OS of MM. Additionally, the risk score model was established based on the two prognostic genes SLC19A1 and LST1. Through further univariate and multivariate Cox regression analyses, we found that the risk score model was well validated in the GSE24080 and GSE4581 datasets. These two genes showed good predictive performance, indicating that SLC19A1 and LST1 may act as independent prognostic factors for MM patients. Overall, our data suggested that genetic overexpression of SLC19A1 is correlated with inferior prognosis in MM.

Previous studies revealed that solute carrier (SLC) transporters play an important role in MM drug resistance, including resistance to novel therapies (44). SLC19A1 belongs to the solute carrier (SLC) group of transporters and is the main mechanism by which folates and antifolate drugs are delivered to mammalian cells and tissues (45). High levels of SLC19A1 transcripts are detected in the liver and placenta, with appreciable levels in other tissues, including bone marrow (46). SLC19A1 was recognized to drive uptake of the new generation antifolate pemetrexed (PMX) into tumor cells (47). Recent studies showed that the mRNA expression levels of SLC19A1 were markedly increased in bladder tumor specimens (25). SLC19A1 was significantly highly expressed in osteosarcoma cells (48). Maggini et al. reported that the SLC19A1 T-233T genotype is associated with an improved response after treatment with melphalan and ASCT in MM. In this study, we identified SLC19A1 as a hypoxia–immune-related gene in MM, which has been found to be the major importer of 2′3′-cyclic-GMP-AMP (cGAMP) and other CDNs (29). cGAMP is an immunotransmitter secreted by cancer cells that, when taken up by host cells, can trigger an antitumoral immune response (29, 49). Due to the antitumoral effects of cGAMP, SLC19A1’s role in MM is worthy of further study. Furthermore, our data suggested that the expression of SLC19A1 in newly diagnosed or relapsed MM patients was significantly increased. Moreover, the upregulation of SLC19A1 has been found to be associated with higher risk stratification, bone marrow aspiration plasma cells, and β_2_-microglobulin level in MM patients.

In conclusion, we established a hypoxia–immune-associated gene risk score model for predicting the survival of MM patients by differential expression analysis and univariate and multivariate Cox regression analyses. The overexpressed gene SLC19A1 may be a prognostic biomarker for the inferior survival of MM patients, which is supported by previous studies (34). In addition, SLC19A1 is a potential hypoxia–immune-related gene for MM, which is really worthy of further study about the function of SLC19A1 in multidrug resistance and the molecular mechanism in hypoxic immune niches.

## Data Availability Statement

The data is publicly accessible in the Gene Expression Omnibus (GEO) and can be found at the website: https://www.ncbi.nlm.nih.gov/geo/, accession numbers (GSE24080 and GSE4581).

## Ethics Statement

The studies involving human participants were reviewed and approved by the Cancer Research Institute Review Board of Central South University (CSU). The patients/participants provided their written informed consent to participate in this study.

## Author Contributions

XW and DJ conceived, designed and supported the study. WJL and PY performed experiments and wrote the manuscript. WJL and QM conducted most of the data analysis. WQL, LX, and CX participated in collecting data and helped in data analysis. SX and YH revised the paper. All authors reviewed and approved the manuscript.

## Funding

This work was supported by the National Natural Science Foundation of China (82060044) and the Natural Science Foundation of Guangxi Province (2020GXNSFAA159018).

## Conflict of Interest

The authors declare that the research was conducted in the absence of any commercial or financial relationships that could be construed as a potential conflict of interest.

## Publisher’s Note

All claims expressed in this article are solely those of the authors and do not necessarily represent those of their affiliated organizations, or those of the publisher, the editors and the reviewers. Any product that may be evaluated in this article, or claim that may be made by its manufacturer, is not guaranteed or endorsed by the publisher.
