# Supplementary figures and images for: Hypoxia–Immune-Related Gene SLC19A1 Serves as a Potential Biomarker for Prognosis in Multiple Myeloma
Source: Front Immunol. 2022 Jul 25;13:843369. doi: 10.3389/fimmu.2022.843369 (PMC9358019; doi:10.3389/fimmu.2022.843369)

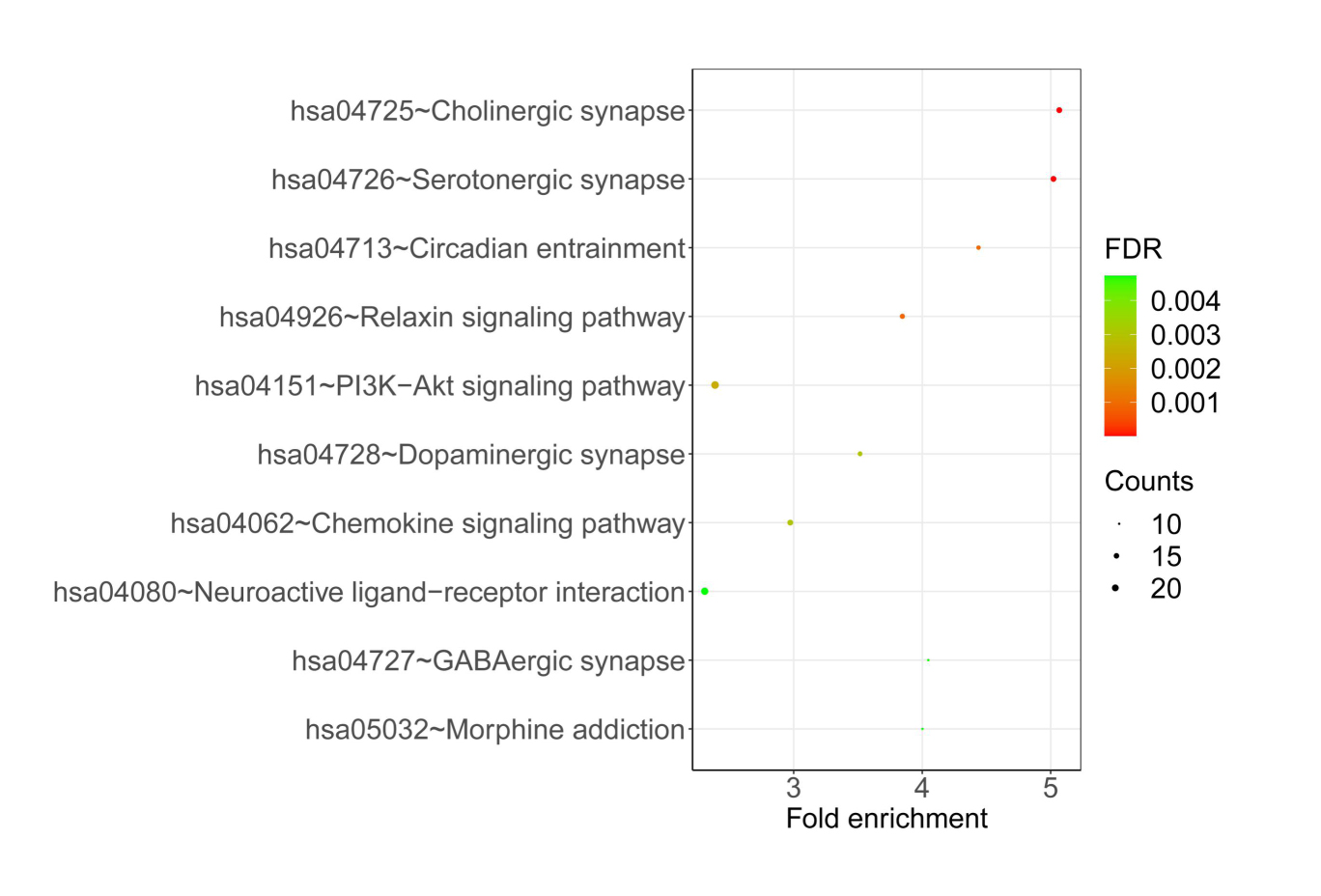


Figure S1. Functional enrichment analysis of the 639 DEGs.

Supplement: Supplementary file 1 [file DataSheet_1.doc]
